# On Lightning Rods and Their Improvement

**Published:** 1840

**Authors:** R. Hare

**Affiliations:** Professor of Chemistry in the University of Pennsylvania


*On the causes of the inadequate protection afforded by Lightning Rods, in some cases, and the means of ensuring their perfect competency; also, a refutation of the prevalent idea that Metals are peculiarly attractive of Electricity; by* R. Hare, M. D., *Professor of Chemistry in the University of Pennsylvania.*

In some of our American newspapers, a letter has been re-published from the London Times, calculated, as I conceive, most perniciously to lessen the confidence of the public in metallic conductors, as a means of protection against lightning. In common with many other persons, the author of the letter appears to suppose, that metals are peculiarly attractive of electricity; and infers that, when a metallic rod is attached to a house, or ship, a discharge of electric fluid may be induced from a cloud, which otherwise would not have been sufficiently near to endanger the premises. Nothing, in my opinion, can be more erroneous than this notion. The truth is, that the earth and the thunder cloud being in opposite electrical states, the electric fluid tends to pass from one to the other, in order to restore the equilibrium. The atmosphere being a non-conductor, through which a discharge cannot be accomplished without a forcible displacement of air, any solid body rising above the earth's surface, which may be more capable than the air of transmitting electricity, is made the medium of communication. Metals being pre-eminently capable of acting as conductors, the transmission of electricity is made through them with proportionably greater facility. Yet they do not attract it more than other substances similarly electrified. A glass, or wooden ball, is as readily attracted, by the excited conductor of an electrical machine, as a ball of metal, and as much more than a metallic point, as the superficies of the ball may be greater than that of the point.

Nothing to me appears more unfounded than an idea, lately suggested, that the attraction between a ship and a thunder cloud, can be increased by the presence of a pointed metallic rod surmounting the main mast.

If houses or vessels have been struck with lightning, while provided with conductors, it is, in my opinion, owing to the conductors being improperly constructed; or having no adequate connection with the earth. The power of any body to receive an electric discharge, is dependent on the conducting power of the medium in which it terminates, no less than upon its own. A metallic rod, held by a glass handle or entering a mass of pounded glass, or dry sand, would not be more efficacious as a conductor, than a glass rod similarly situated. If terminated by an imperfect conductor, as for instance by earth or water, its power is reduced in proportion to the imperfection of the medium thus bounding it. This influence of the media, in which conductors terminate, has not been sufficiently insisted upon in treatises on electricity. I should not consider a metallic rod, terminating without any enlargement of surface, in the water or the earth, as an adequate protection against lightning: but were such conductors to terminate in metallic sheets, buried in the earth or immersed in the sea, or *by a connection duly made with the iron pipes, with which our city is watered, or the copper with which ships are generally sheathed*, I should have the most perfect confidence in their competency.

It is not only important that the points of contact between the metallic mass employed to afford lightning an adequate passage, and the earth or water in which it terminates, should be so multiplied as to compensate for the inferior conducting power of the earth or water; but it is also necessary that the conducting rod be as continuous as possible. When conductors are to be stationary, as when applied to buildings, they shonld consist of pieces screwed together, or preferably, joined by solder, as well as by screwing. Where flexibility is requisite, the joints should be neatly made, like those of the irons of fall-top carriages; and should be riveted so as to ensure a close contact at the junctures.

In all cases the ordinary, but important precaution of having the rod to terminate above, in a fine clean point, should be attended to. Where platina tips cannot be had, multiplying the points by splitting the rod into a ramification of pointed wires. may compensate for the diminution of conducting power, arising from rust.

The efficacy of the point or points, is, however, dependent on the continuity of the conductor of which I have already spoken; since it is well known, that if a pointed rod be cut into parts, so as to produce intervals, bounded by blunt terminations, its efficicacy will not be much greater than if it had no point; because the fluid will, in that case, pass in sparks, instead of being transmitted in a current. It is on this account that I object to chains, or rods joined by loops or hooks and eyes. The error of supposing that a metallic rod must be more capable of attracting electricity injuriously, because of its known wonderful power in transmitting it, will be evident when it is understood that the only difference between metals and other bodies, arises from the superior power of transmission. Hence, when by a defective communication with the earth or sea, the efficacy of the metal, as a conductor, is diminished, or destroyed, its influence over a charged cloud is proportionably lessened. It follows, therefore, that so far as it acts, its action must be beneficial, unless its lower termination should, by an inconceivable degree of ignorance or inattention, be so situated as to render it more easy for the electric fluid to leave the rod, and pass through a portion of the house or vessel, than to proceed, by means of the rod, into the earth or sea.

Thus, Richman was killed by a conductor which he employed to receive electricity from the clouds, and to convey it to an electrometer, necessarily insulated: under these circumstances, the head of the professor being about a foot from the conductor, he became a part of the channel of communication with the earth. Had the apparatus been surrounded by a cage of wire, and this duly connected with a metallic rod soldered to a sheet of metal buried in the earth, Richman might have made his observations with perfect security. That, with due precaution, experiments analogous to his, are not productive of injury to the operatior, is rendered evident by the subjoined quotation from Singer's Electricity.

I must premise, that the apparatus, by means of which the phenomena alluded to were produced, consisted of a wire a mile long, supported and insulated, upon very high poles, and terminating in the house of the electrician, Andrew Crosse, Esq.

"The approach of a charged cloud, produces sometimes positive, and at others negative signs, at first; but, whatever be the original character, the effect gradually increases to a certain extent, then decreases, and disappears, and is followed by the appearance of the opposite signs, which gradually extend beyond the former maximum, then decrease, terminate, and are again followed by the original electricity. These alternations are sometimes numerous, and are more or less rapid on different occasions; they usually increase in intensity at each repetition, and at last a full dense stream of sparks, issues from the atmospherical conductor to the receiving ball,* stopping at intervals, but returning with redoubled force. In this state a strong current of air proceeds from the wire and its connected apparatus; and none but a spectator can conceive the awful, though sublime ,effect, of such a phenomenon. At every flash of lightning, an explosive stream, accompanied by a peculiar noise, passes between the balls of the apparatus, and enlightens most brilliantly, every surrounding object, whilst these effects are heightened by the successive peals of thunder, and by the consciousness of so near an approach to its cause.

"During the display of electric power, so awful to an ordinary observer, the electrician sits quietly in front of the apparatus, conducts the lightning in any required direction, and employs it to fuse wires, decompose fluids, or fire inflammable substances; and when the effects are too powerful to attend to such experiments securely, he connects the insulated wire with the ground, and transmits the accumulated electricity in silence and with safety."

---

* That is, a ball communicating with the earth, by an adequate metallic conductor.

